# SEED Servers: High-Performance Access to the SEED Genomes, Annotations, and Metabolic Models

**DOI:** 10.1371/journal.pone.0048053

**Published:** 2012-10-24

**Authors:** Ramy K. Aziz, Scott Devoid, Terrence Disz, Robert A. Edwards, Christopher S. Henry, Gary J. Olsen, Robert Olson, Ross Overbeek, Bruce Parrello, Gordon D. Pusch, Rick L. Stevens, Veronika Vonstein, Fangfang Xia

**Affiliations:** 1 Computation Institute, University of Chicago, Chicago, Illinois, United States of America; 2 Mathematics and Computer Science Division, Argonne National Laboratory, Argonne, Illinois, United States of America; 3 Fellowship for Interpretation of Genomes, Burr Ridge, Illinois, United States of America; 4 Department of Computer Science, San Diego State University, San Diego, California, United States of America; 5 Department of Microbiology and Immunology, Faculty of Pharmacy, Cairo University, Cairo, Egypt; 6 Department of Microbiology, University of Illinois at Urbana-Champaign, Urbana, Illinois, United States of America; 7 Computing, Environment, and Life Sciences, Argonne National Laboratory, Argonne, Illinois, United States of America; University of Hyderabad, India

## Abstract

The remarkable advance in sequencing technology and the rising interest in medical and environmental microbiology, biotechnology, and synthetic biology resulted in a deluge of published microbial genomes. Yet, genome annotation, comparison, and modeling remain a major bottleneck to the translation of sequence information into biological knowledge, hence computational analysis tools are continuously being developed for rapid genome annotation and interpretation. Among the earliest, most comprehensive resources for prokaryotic genome analysis, the SEED project, initiated in 2003 as an integration of genomic data and analysis tools, now contains >5,000 complete genomes, a constantly updated set of curated annotations embodied in a large and growing collection of encoded subsystems, a derived set of protein families, and hundreds of genome-scale metabolic models. Until recently, however, maintaining current copies of the SEED code and data at remote locations has been a pressing issue. To allow high-performance remote access to the SEED database, we developed the SEED Servers (http://www.theseed.org/servers): four network-based servers intended to expose the data in the underlying relational database, support basic annotation services, offer programmatic access to the capabilities of the RAST annotation server, and provide access to a growing collection of metabolic models that support flux balance analysis. The SEED servers offer open access to regularly updated data, the ability to annotate prokaryotic genomes, the ability to create metabolic reconstructions and detailed models of metabolism, and access to hundreds of existing metabolic models. This work offers and supports a framework upon which other groups can build independent research efforts. Large integrations of genomic data represent one of the major intellectual resources driving research in biology, and programmatic access to the SEED data will provide significant utility to a broad collection of potential users.

## Introduction and Motivation

The growth in the number of sequenced genomes has exceeded the most ambitious past estimations [1], [2]. Today, tens of thousands of genomes are being sequenced by public and private institutions around the globe [3], [4], and whole-genome sequencing/re-sequencing has become a routine step of virtually any analysis of microbial behavior. In some fields, researchers are sequencing hundreds of genomes from distant taxa [5] to explore microbial diversity, while elsewhere, hundreds of closely related genomes are being sequenced to understand environmental adaptation, to support pangenome reconstruction [6], [7], [8], and to build maps of microbial variomes [9]. Sequencing is also being applied to aid in solving urgent biological problems, including the development of strategies to combat emerging or reemerging biothreats, such as the severe acute respiratory syndrome (SARS) virus [10], the 2009 H1N1 influenza virus [11], [12], and the 2011 German *Escherichia coli* outbreak [13], [14].

With the vast speed and minimal costs of modern sequencing technology, new demands are being placed on the bioinformatics pipelines used to analyze sequence data [1]. Modern bioinformatics must be high throughput and high volume with a strong emphasis on comparative genomics, and the SEED family of resources for genome interpretation and analysis were specifically constructed to embody these characteristics [15]. The SEED resources are built upon the organization of biology into a modular set of subsystems, each describing a specific biological functionality (e.g., flagellar motility or histidine biosynthesis) [16]. Subsystems-based technologies were developed in the SEED with the view that the interpretation of one genome can be made more efficient and consistent if hundreds of genomes are simultaneously annotated in one subsystem at a time [16], [17]. The SEED Project, a multi-institutional effort coordinated by the Fellowship for Interpretation of Genomes (FIG) and Argonne National Laboratory, began with the ambitious goal of consistently annotating 1,000 genomes (http://theseed.org/wiki/Annotating_1000_genomes); today, over 50,000 viral and prokaryotic genomes have been annotated by the subsystems technology. More recently, the SEED Project has been expanded to include the capacity to automatically generate genome-scale metabolic models based on SEED annotations of genome sequences, and the Model SEED system developed from this pipeline has now been applied to construct metabolic models for thousands of genomes [18], [19], [20]. The SEED family of resources now collectively serves as a repository for almost 5,000 distinct complete prokaryotic genomes, associated with approximately 30,000 annotations, 11,000 metabolic models, 178,000 protein families, 10,250 functional roles, and 1,060 subsystems.

While a web interface is available for visualizing the data within the SEED (http://pubseed.theseed.org), this interface is inadequate to make full effective use of the massive quantities of data now embodied within the SEED. To truly expose the SEED data for efficient large-scale use by the global scientific community, we initially developed a Simple Object Access Protocol (SOAP) server to allow the needed programmatic access to the SEED data [21]; however, the SOAP server could not scale up with the increased volume of data. To cope with the increasing demands placed on the SEED web services, as well as the increasing volumes of data included within the SEED resources, we developed a second-generation of web services to improve throughput, response time, and access to SEED functionality. Here, we describe in detail the four primary servers now available for remotely accessing SEED data and we describe the client-side distribution of server scripts that we provide as examples of how the servers may be applied to perform complex analyses of genome data.

## Design and Implementation

### The Servers and the Services

**Figure 1:**
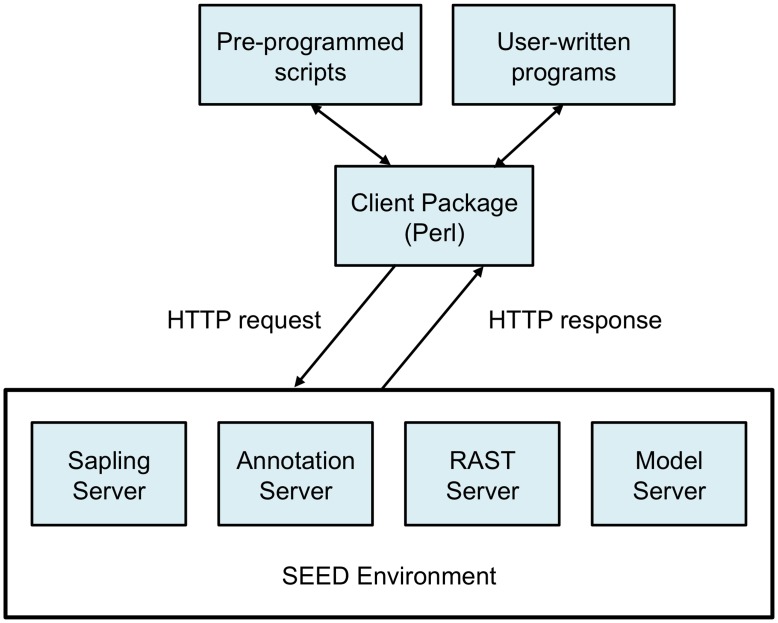
Architecture of the SEED servers. The client packages (currently available for Perl or Java) handle the HTTP requests and responses, and parse the data from the appropriate lightweight data exchange formats to data structures. The four servers access the SEED data.

The four servers described here, collectively referred to as the SEED servers, currently support 181 methods that can be invoked to extract data and services (Fig. 1). The server code resides at the location of the SEED data. Users download a distribution with their choice of runtime environment that they may use to write programs to access SEED data or perform a number of common bioinformatics tasks using a supplied set of preprogrammed scripts. Currently, Perl and Java integrations are supported. The following subsections describe the four servers in more detail.

### 1. The Sapling Server

The *Sapling Server* offers access to the underlying integration of genomic data–including genomes, genes, proteins, annotations, subsystems, FIGfams, and co-occurrence data. On the server side, the *Sapling Server* accesses a database implemented by an entity-relationship data model (ERDB). The ERDB model is defined by a set of XML metadata describing the entities, relationships, and attributes in a form that can be used to generate queries as well as the documentation and an up-to-date database diagram may be viewed at http://servers.nmpdr.org/figdisk/FIG/ErdbDocWidget.cgi?database=Sapling. Thus, the public description of the database always remains synchronized with the internal data structures–an important benefit in a database designed for public use.

The *Sapling Server* is architected such that new features can be added quickly. New data tables may be added as updates to the XML metadata, which is processed by a special load program to build the initial database tables. The list of services offered is maintained on the server, so that client software does not need to be updated in order for users to access new features. A web application that converts general database queries to Perl code helps speed implementation of new functions.

**Figure 2:**
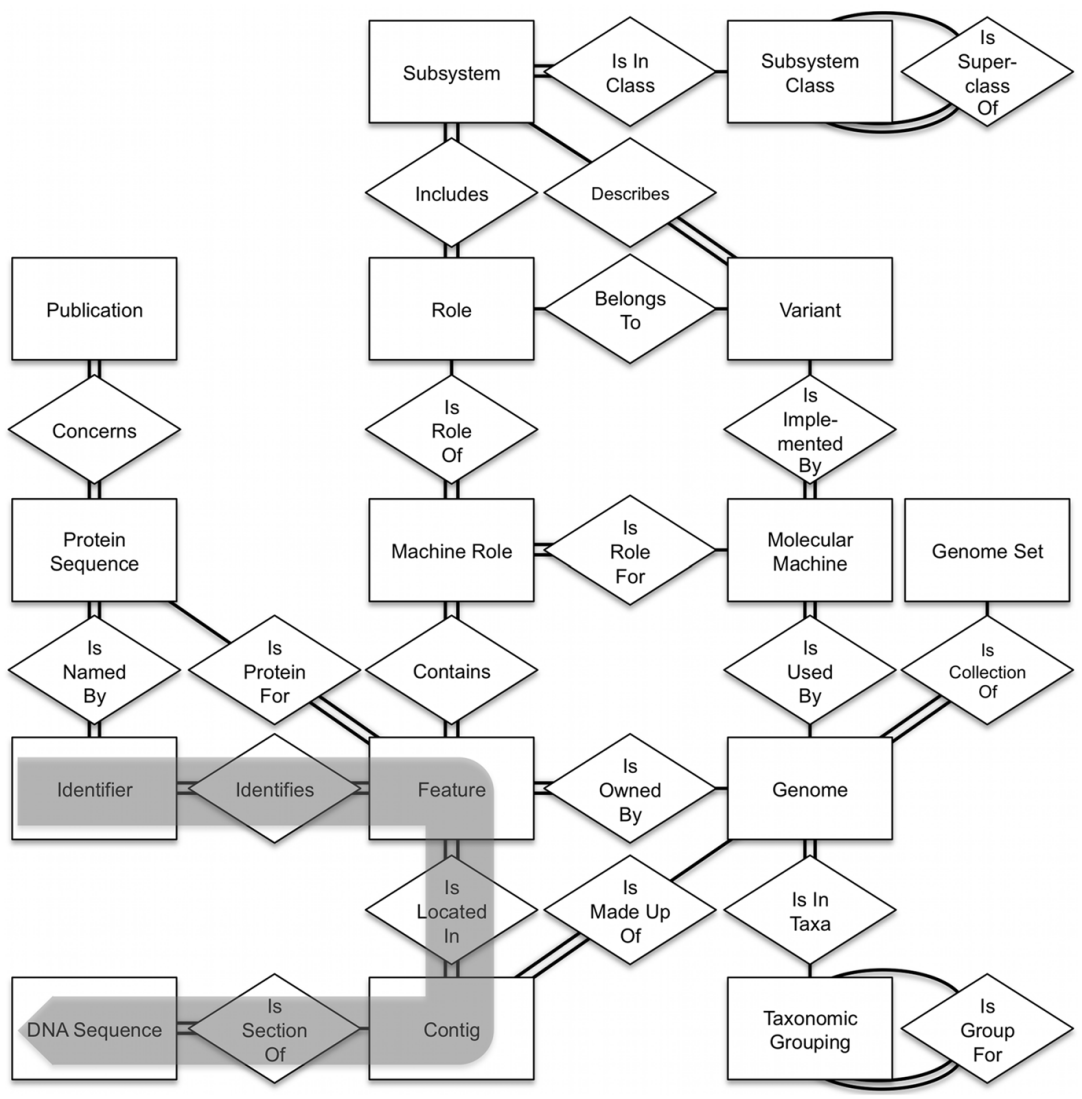
Entities and relationships in the SEED. The entities (boxes) are connected to each other by a series of relationships (diamonds) that describe how the two entities relate. To move from one entity (e.g., “Identifier”) to another (e.g., “DNA Sequence”), the series of connections shown by the gray arrow is made. This way, any entity can be connected, either directly or indirectly, to any other entity.

A database query is specified by naming the entities and relationships along a path through the ERDB diagram (Fig. 2) along with a list of the data items to be returned and a filter clause that limits the results to the desired data objects (e.g., a particular genome or identifier). The *Sapling Server* allows direct queries against the database; however, a set of common data requests is implemented as direct server functions. *Sapling Server* functions typically accept multiple input values within a single call, allowing a client to minimize the number of requests that must be made to the server. Additional input parameters allow a client to modify the query, for example, to request that the output be in FASTA format or to ask for protein rather than DNA sequences.

**Figure 3:**
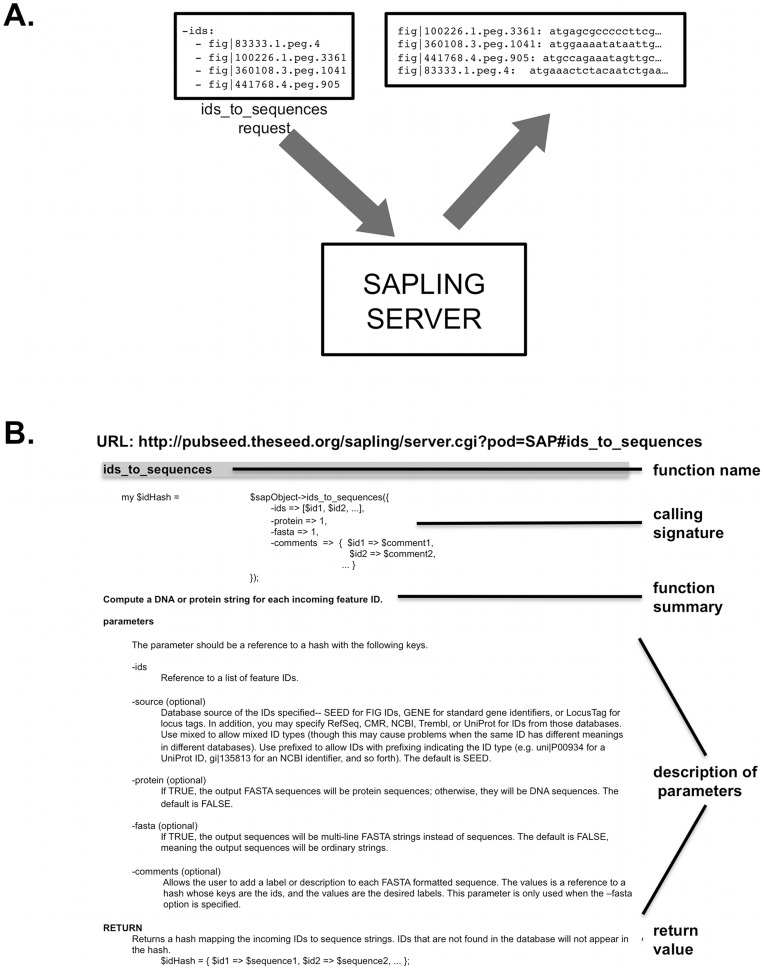
Processing ids_to_sequences. (a) The ids_to_sequences function call accepts multiple IDs as an argument and uses the Sapling server to process the calls. These are returned as a single table. (b) A detailed description of each call (in this example, the ids_to_sequences) is provided online and is automatically generated from the entity-relationship models shown in Figure 2.

In a sample **ids_to_sequences** request (Fig. 3A), the user specifies four identifiers, and the server returns them as a table (actually a Perl hash) with the associated DNA sequences attached.

The *Sapling Server* currently supports 127 access functions. These functions are listed on a web page generated automatically from the latest version of the code, ensuring that the documentation remains up to date. A sample showing the web page description of **ids_to_sequences** is shown (Fig. 3B).

### 2. The Annotation Support Server

The *Annotation Support Server* supports two distinct capabilities relating to the annotation of genomes: *de novo* annotation of protein or DNA sequences, and aggregation of annotations into subsystems. The *Annotation Support Server* accepts either DNA or protein sequence as input and, depending on the user options, can either use existing gene calls or invoke standard gene callers (e.g., *GLIMMER-3* for protein-encoding genes). The server also houses newly developed high-performance methods to assign function to protein sequences or regions of genomic DNA sequences, based on FIGfams [22]. Below is an example application using these methods that produces a relatively accurate annotation of most microbial genomes within a few minutes. To evaluate the technology, users are encouraged to simply submit a known prokaryotic genome to the server for annotation.

Sequences can be submitted to the server in three ways:

1. Programs can directly access the services needed to call genes and assign functions to the proteins encoded within the genome.
2. If the protein-encoding genes have already been identified, the program can assign functions to these sequences. An example program is provided in the download library and is described at http://servers.theseed.org/sapling/server.cgi?pod=svr_assign_using_figfams.pl.
3. A program can take as input fragments of DNA (e.g., from a metagenomic sample) and use the services to detect pieces of protein-encoding genes. Again, an example program is provided in the download library and is described at http://servers.theseed.org/sapling/server.cgi?pod=svr_assign_to_dna_using_figfams.pl).

The *Annotation Support Server* also provides the ability to take as input a set of functional roles (in the controlled vocabulary established by the subsystem collection) and to produce a detailed estimate of which subsystems are represented by those functional roles. That is, one can also use the server to develop a metabolic reconstruction based on the functional roles that have been assigned to the protein-encoding genes.

### 3. The RAST Submission/Retrieval Server

The RAST (Rapid Annotations using Subsystems Technology) [23] service provides access to high-throughput, high-quality annotation of prokaryotic genomes, and routinely processes ∼25–75 genomes a day, with peak throughputs that have exceeded 200 genomes per day. This service is accessible for individual genome submission via a graphical web interface, and the next generation SEED servers provide new access for programmatic batch submission of genomes via the RAST submission and retrieval server. This server supports programmatic submission of genomes to the *RAST Server*, retrieval of job status, and retrieval of the final set of annotations. These scripts and the underlying application programming interface (API) enable users to submit genomes to the *RAST Server*, test the status of submitted jobs, and retrieve the output (i.e., annotated genomes).

Three types of input are supported:

1. A FASTA file of contigs that make up the genome to be annotated
2. A file of GenBank-formatted entries (with the option to retain the gene calls as given in the uploaded files)
3. An Entrez ID or a Genome Project ID.

In the case of the first type of input (FASTA file), the *RAST Server* will perform *de novo* annotation, i.e., will start by calling RNA- and protein-coding genes, and then will proceed to functional annotation, subsystems assignment, and subsequent metabolic reconstruction. In the second case (GenBank-formatted files), gene calling is optional, since GenBank-formatted files already include the coordinates for open-reading frames and RNA genes. In the last case (Entrez ID or Genome Project ID), the tools provided within the *RAST Submission/Retrieval Server* will query NCBI using the query ID(s) and will retrieve the sequence (whether that sequence consists of one or a set of contigs that make up the genome project); that sequence then becomes the input to the *RAST Server*.

### 4. The Metabolic Modeling and Flux Balance Analysis Server

The *Metabolic Modeling and Flux Balance Analysis (FBA) Server* provides programmatic remote access to the Model SEED biochemistry and genome-scale metabolic model database, as well as some model analysis algorithms. The Model SEED biochemistry database integrates into a single, nonredundant set all the reactions and compounds found in the Kyoto Encyclopedia of Genes and Genomes (KEGG) database [24], [25], [26], together with additional curated reactions and compounds [18], and a continuously growing number of published genome-scale metabolic models.

Currently this database consists of 16,279 compounds and 13,272 reactions. For compounds, the database also includes database IDs from KEGG compounds and SEED models, names/synonyms, molecular masses, molecular formulas, molecular charge, and estimated Gibbs free energy of formation [27]. For reactions, the database includes database IDs from KEGG reactions and SEED models, names/synonyms, stoichiometry, EC numbers, pathways, and estimated Gibbs free energy change of reactions [27]. Compound charge, formula, formation energies and reaction stoichiometry are all calculated for aqueous conditions at neutral pH. All API functions used to access the *Metabolic Modeling and FBA Server* capabilities are documented in detail at http://servers.theseed.org/.

The SEED database also contains a large number of genome-scale metabolic models, including 14 published models [28] and approximately 11,000 models generated from the annotated genomes stored in the SEED [29]. The *Metabolic Modeling and FBA Server* also provides the user with an API to remotely obtain a list of the models in the SEED and to download data on the compounds and reactions in each SEED model. The server returns the following data for each reaction in a specified model: (i) all data from the SEED biochemistry database, (ii) a list of the genes associated with each reaction in the model in a format that captures how the protein products encoded by the genes function to catalyze the reaction (as either independent enzymes or multienzyme complexes), and (iii) a list of compartments in the model where the reaction takes place and the directionality/reversibility of the reaction in each compartment. For the model compounds, the server returns the data from the SEED biochemistry database. As with the biochemistry data, all the model data in the server are accessible either via Perl programs or the API.

The *Metabolic Modeling and FBA Server* also enables users to run various FBA studies on any of the genome-scale metabolic models stored in the SEED database. These studies can be performed while simulating any of 525 distinct media conditions currently encoded in the SEED database (which includes all Biolog® [30] media conditions and a variety of complex media formulations). Both the Perl program and the API enable users to obtain a list of the media conditions currently stored in the SEED and details on the compounds included in each formulation. Once a model and media condition have been selected for simulation, the server provides an interface for running three types of FBA simulation: (i) *simple growth simulation* to predict maximum growth rate of the organism in the selected media, (ii) *flux variability analysis* (FVA) [31] to classify the reactions and compounds in the model according to their behavior during growth in the selected media, and (iii) *single gene knockout analysis* to predict the genes essential for growth in the selected media.

The simple growth simulation returns the maximum predicted growth rate of the model given the input parameters, the predicted flux through the model reactions during maximum growth, and the predicted uptake and production of nutrients from and to the environment during maximum growth.

The FVA simulation returns the predicted class of every reaction and compound in the model during growth given the input parameters. Reactions in the model are classified as *forward essential* or *reverse essential* if they are required for growth to occur, with *forward* and *reverse* referring to the direction in which the reactions must proceed. Reactions that are not essential for growth, but still active, are classified as *forward variable*, *reverse variable*, and *variable*, with *forward* and *reverse* indicating when reactions proceed only in a single direction. Reactions are classified as *blocked* if they cannot carry flux under the conditions specified by the user. Metabolites in the model are classified as *essential nutrients* or *essential products* if their uptake or secretion is required for growth in the input conditions, and they are classified as *transported* if they can be taken up or secreted but are not essential for growth. In addition to classifying the reactions and compounds in the model, the FVA simulation returns the maximum and minimum values for the flux through each reactions and the uptake/secretion of each metabolite.

The single gene knockout analysis rapidly simulates the individual knockout of every gene represented in the model during growth in the input conditions. Based on these simulations, the analysis produces a list of the predicted essential genes and the predicted nonessential genes in the model. Both the Perl program and API allow the user to run any of the three simulation types from the command line.

All three simulation types accept the same user input: the name of the model to be run, the name of the media formulation that growth should be simulated in, a list of genes in the model that should be knocked out during the simulation, and a list of the reactions in the model that should be knocked out during the simulation. See http://servers.theseed.org/for detailed documentation on all *Metabolic Modeling and FBA Server* functions.

## Applications

The SEED servers can have limitless applications. Below, we demonstrate various applications of SEED servers through a small set of potential applications and coding examples.

### Using SEED Servers to Convert Gene and Protein IDs

Dealing with IDs of genes or the proteins they encode is often nontrivial. In the SEED database, we use IDs that specify protein-encoding genes in a rapidly growing set of genomes, and we support correspondences between these IDs and those used by other annotation efforts. The SEED has two notions of equivalence: (i) two IDs that represent either protein-encoding genes or protein sequences are said to be *sequence equivalent* if the protein sequences are identical and (ii) two IDs that represent either exactly the same protein-encoding gene or the precise protein encoded by the gene (that is, “the protein sequence of gene *X* in genome *Y*”) are said to be *precisely equivalent*. Unfortunately, in the presence of multiple versions of thousands of genomes, perfect maintenance of the “precisely equivalent” correspondence is virtually impossible.

Our first example script takes a command-line argument containing a single ID and produces a table for all assertions of functions for sequence equivalent IDs. Each ID in the input is associated with the name of the genome containing it, the function for that ID, the source of the functional assignment assertion, and an indication of whether the source of the assertion provided a confidence for their estimate. The code is available at http://servers.theseed.org/sapling/server.cgi?code=server_paper_example1.pl (also provided as Supporting Information S1).

### Using SEED Servers to Generate a Metabolic Reconstruction

Given a set of functional roles, one often wishes to understand which subsystems can be inferred from the set. The following example script reads as input a set of functional roles and constructs a table of subsystems that can be identified, along with their variation codes. The data displayed in this simple example could form the start of a research project to gather the functional roles not connected to subsystems to determine whether they were not connected because a small set of functional roles were not present in the input, and to seek candidates for such "missing functional roles." The ability to easily map functional roles into subsystems will improve as the SEED annotation effort improves its collection of encoded subsystems [32]. The code for this example is shown at http://servers.theseed.org/sapling/server.cgi?code=server_paper_example2.pl. (also provided as Supporting Information S1).

### Using SEED Servers to Create Custom Interfaces

The SEED provides the ability to graphically display the chromosomal regions around a set of genes (normally from distinct genomes); for example, see http://seed-viewer.theseed.org/seedviewer.cgi?page=Annotation&feature=fig|83333.1.peg.4. The SEED also offers an alternative for creating custom interfaces, moreover, one that does not require the user to know appropriate SEED IDs. This approach exploits the conversion capabilities of the SEED for creating a program to accept arbitrary protein IDs. It also exploits the ability of SEED to map functional roles into subsystems as described in the preceding example. The result is a tool that enables the user to take a SEED ID and a region size, and extract the genes that are found within a region centered on the designated gene. The code for this example is shown at. http://servers.theseed.org/sapling/server.cgi?code=server_paper_example3.pl (also provided as Supporting Information S1).

### Using SEED Servers to Analyze Chromosomal Co-occurrence through Access to Functional Coupling Data

A great deal has been learned from studying genes that tend to occur close to one another in diverse genomes [33], [34], [35], [36], [37], [38]. In particular, the co-occurrence of hypothetical and non-hypothetical proteins can be exploited to suggest the function of the former based on the function of the latter.

The following program at http://servers.theseed.org/sapling/server.cgi?code=server_paper_example4.pl illustrates the potential for constructing custom tools by going through all of the protein-encoding genes in all of the complete prokaryotic genomes maintained within the SEED looking for “hypothetical proteins” that tend to co-occur with genes encoding functions that can be connected to subsystems. The program constructs a table showing the following:

- Gene
- Function of the gene
- Genome id containing the gene
- Biological name of the genome
- Non-hypothetical gene in a subsystem that appears to have the strongest measure of co-occurrence
- Measure of gene co-occurrence
- Function assigned to the co-occurring gene contained in a subsystem.

This table can therefore be used to suggest functions for hypothetical proteins that could be tested experimentally. A copy of the code is provided (Supporting Information S1).

### Using SEED Servers to Assign Functions to a Set of Protein Sequences

The SEED can be used to assign functions to a file of protein sequences. The code for this example is at http://servers.theseed.org/sapling/server.cgi?code=server_paper_example6.pl (also provided as Supporting Information S1).

This program reads a FASTA file of protein sequences and attempts to assign function to those sequences using a K-mer–based algorithm. When a function is proposed, the program will produce a “score” (the number of distinct K-mers that were matched) and an estimate of phylogenetic neighborhood–a representative genome that is “phylogenetically close” to the genome containing the protein, if an estimate can reasonably be given.

A similar approach has been adopted for rapid, real-time analysis of metagenomics samples, which might elsewhere take days or months for BLAST-based analysis. This real-time metagenomics analysis (URL: http://edwards.sdsu.edu/rtmg) can be performed on computers or cellular phones [39].

### Using SEED Servers for Running FBA on the SEED Model of *Escherichia coli*

Here, we demonstrate how to run a variety of FBA algorithms on the SEED model of *E. coli* and how to print all data from the *E. coli* model and the results of the FBA into an output table. For the code, see http://servers.theseed.org/sapling/server.cgi?code=server_paper_example7.pl (also provided as Supporting Information S1).

The program starts by obtaining a list of all compounds and reactions in the SEED *E. coli* model (Seed83333.1) using the “*get_compound_id_list*” and “*get_reaction_id_list*” functions, respectively. The program then uses these lists to obtain detailed data on all the *E. coli* compounds and reactions (using the “*get_compound_data*” and “*get_reaction_data*” functions, respectively). These data are stored in two tables: one for compounds and one for reactions. Next the “*classify_model_entities*” function is used to run a FVA on the SEED *E. coli* model. In this particular FVA, the reactions and compounds in the *E. coli* model are classified while simulating growth in LB media (called ArgonneLBMedia in the SEED model). At this point, the data returned by the “*classify_model_entities*” function is added onto the compound and reaction tables prepared previously. In the next step, the code uses the “*simulate_model_growth*” function to run a standard FBA on the SEED *E. coli* model, maximizing the model growth rate in simulated glucose minimal media (called Carbon-D-Glucose in the Model SEED). The data returned by this function are also added to the reaction and compound tables. In the final call to the server, the program uses the “*simulate_all_single_gene_knockout*” function to simulate the single knockout of all *E. coli* genes, and the results of this study are stored in a gene table. The remainder of the program handles the printing of the compound, reaction, and gene tables to the files CompoundTbl.txt, ReactionTbl.txt, and GeneTbl.txt, respectively.

### Using SEED Servers for Mapping SEED to Gene Ontology Functions

The Gene Ontology (GO) project aims to unify biology by providing a controlled vocabulary of terms for all genes and gene products [40], but has long had a focus on eukaryotes with less emphasis on prokaryotes and their viruses. On the other hand, the SEED database contains high quality annotations for hundreds of microbial and viral genomes, using subsystems-based controlled vocabulary. Mapping SEED functional roles to GO annotations for a given set of genes or gene products can be achieved via SEED servers. A workflow, detailed elsewhere (Short URL: http://bit.ly/server_paper_example8 ), uses two SEED servers-based programs to update SEED to GO comparisons through the use of UniProt [41], [42] protein identifiers.

The workflow consists of the following steps:

1. Download and install the SEED servers from http://www.theseed.org/servers/#mozTocId509100
2. Download the latest version of UniProt: ftp://ftp.uniprot.org/pub/databases/uniprot/current_release/knowledgebase/complete/uniprot_sprot.dat.gz
3. Download the parser: http://edwards-sdsu.cvs.sourceforge.net/viewvc/edwards-sdsu/bioinformatics/bin/parse_uniprot.pl?view=log (This code is also provided in Supporting Information S1)
4. Run the parser on the UniProt file: perl parse_uniprot.pl uniprot_sprot.dat.gz > uniprot_md5_go_prot.txt
5. Download the code to map the parsed UniProt file to the SEED: http://edwards-sdsu.cvs.sourceforge.net/viewvc/edwards-sdsu/bioinformatics/bin/map_uniprot_to_seed.pl?view=log (This code is also provided in Supporting Information S1)
6. Run the code to compare UniProt to SEED: perl map_to_seed.pl -f uniprot_md5_go_prot.txt > uniprot_seed.txt
7. Compare the results in uniprot_seed.txt

## Discussion

The SEED project [15] focuses on developing technology to support rapid, high-volume, accurate annotation of genomes, and has so far achieved four advances of central importance:

1. The *subsystems strategy*, adopted as the guiding principle of the effort [16], centers on leveraging expert annotations to define a small set of functional roles in all genomes rather than all the functional roles in a small number of genomes.
2. The subsystem effort provided a convenient framework for the curation of a set of protein families that became known as *FIGfams* [22], [23], intended to contain only *isofunctional homologs–*that is, each family was intended to contain only homologous proteins playing the same functional role. When errors in FiGfams are detected, the underlying subsystems are updated and then the FIGfams are regenerated to correct those errors. The rapid evolution of the FIGfam collection has made possible a number of the services described in this article.
3. Using subsystems and FIGfams as the underlying technology, the RAST server was developed and made available in 2007 [23]. Thousands of viral and prokaryotic genomes have been annotated with the RAST system, and hundreds more are being annotated each week.
4. The high-quality annotations generated by RAST together with the ability to manually modify those annotations by human experts allowed for the automatic generation of genome-scale metabolic models in the Model SEED [18], [19], [20].

As indicated above, the improvement in sequencing speed and efficiency led to a rapid accumulation of genome sequences and necessitated more efficient methods for large-scale access to genomes, annotations, subsystems, and genome-scale models within the SEED database. A SOAP server was first developed to allow programmatic access to the SEED data [21], but several performance issues prevented the service from scaling with the volume of data contained within the SEED database. The server abstraction layer required the loading of numerous large modules on each invocation of server functionality, resulting in a noticeable delay in response to each server request. The encapsulation of the results in SOAP XML conferred significant overhead on the data being transferred. Finally, each operation of the SOAP server was atomic, accepting a single argument and returning a single datum. Trivial requests such as retrieving all the functions for all of the proteins in a genome took unacceptably long to complete, requiring a separate call for each protein and instantiating many threads on the server.

The four SEED servers described here provide programmatic access to the SEED data and methods. They expose the current data in a form that is conveniently accessed computationally. The installation and maintenance of the client-side software require minimal effort. We have constructed the underlying methods to support relatively large-grained data transfers, allowing the construction of relatively efficient programs. In comparison to the SOAP server [21], the new web services provide access to larger amounts of data in less time, and they have been engineered to respond to server requests with little or no server-side delay. Furthermore, the new web services provide a more efficient and flexible computing approach because they are designed to process batches of requests at a time, streaming the responses as they complete. These services provide access to the integrated genomic data, subsystems, FIGfams, co-occurrence data, annotation services, RAST annotation submission and job retrieval (thereby offering access to our continuing improvements in microbial annotation), and metabolic modeling. All client modules, code examples and documentation are available online at http://servers.theseed.org, and we are continually expanding these services and improving the underlying documentation.

In conclusion, the SEED integration of genomic data now contains over 5,000 complete or nearly complete genomes, a constantly updated set of curated annotations embodied in a large and growing collection of encoded subsystems, and a derived set of protein families. The client–server code discussed in this article gives users easy programmatic access to the data in the SEED. It has already been successfully been used for building other applications, such as finding prophages in microbial genomes [43], desktop-based RAST genome annotation and comparison (manuscript in preparation), and real-time annotation of metagenomes (RTMG, URL: http://edwards.sdsu.edu/rtmg [39]). We encourage researchers to use this software to retrieve and analyze data from the SEED database and we believe that the underlying implementation of these new servers is efficient enough to address the needs of most users and we will continue providing occasional stand-alone versions of the SEED to users who need more performance or privacy.

## Availability and Future Directions

The latest documentation and downloads are available at the following web addresses: http://servers.theseed.org and http://blog.theseed.org/servers/

- **Project name:** SEED Servers
- **Project home page:** http://www.theseed.org/
- **Operating system(s):** Mac OS, Linux
- **Programming language:** Perl, JAVA
- **License:** SEED Toolkit Public License
- **Any restrictions to use by non-academics:** no limitations

### Distribution of the Server Packages

The SEED servers project is documented and can be downloaded from the servers’ web site, http://servers.theseed.org.

**The Perl distribution contains the following:**

**Client Packages.**

1. The Sapling server - SAPserver.pm
2. The MODEL server - MODELserver.pm
3. The Annotation Support Server - ANNOserver.pm
4. The RAST server - RASTserver.pm

**Programming using the servers.** The SEED servers provide all necessary network operations in a client package that can be used to access the server functions. One uses these like any other Perl package. For instance, to find all genomes in the SEED, one does the following:

#!/usr/bin/perl w

use strict;

use SAPserver;

my $sapObject  =  SAPserver->new();

my $genomes  =  $sapObject->all_genomes();

foreach my $g (sort { $genomes->{$a} cmp $genomes->{$b} }

keys(%$genomes)) {

print "$g\t$genomes->{$g}\n";

}

The function call $sapObject->all_genomes() marshals the correct server-side function call and arguments into a network package, transmits that package to the server, waits for and retrieves the answer, processes any returned error codes, decodes the return package into a Perl data structure, and returns the result. All function calls in all the client packages perform these basic services.

### The Java Distribution Contains the Following

#### Client Packages

The org.theseed.servers.serverConnections package handles connecting to the server, transmitting and receiving the data, and converting data structures from the server into Java data structures. The classes in org.theseed.servers.servers packages handle connecting to each of the servers and making the appropriate calls.

#### Programming using the servers

We recommend that the code be accessed in eclipse (http://www.eclipse.org/), netbeans (http://www.netbeans.org/), or a similar graphical integrated development environment (IDE). These are used like any other class. For instance, to find all genomes in the SEED, one does the following

import java.util.HashMap;

import org.theseed.servers.SAPserver;

public class AllGenomes {

public static void main(String [35] args) {

SAPserver sapling  =  new SAPserver();

HashMap<String, String>genomes  =  sapling.allGenomes();

for (String id : genomes.keySet()) System.out.println(id + "\t"+ genomes.get(id));

}

}

Future directions include expanding the applications and releasing packages for use by other programming languages such as Python.

## Supporting Information

Supporting Information S1 **Examples of programming using the SEED servers (coded in Perl).** Eight application examples are provided, seven of which (1–4 and 6–8) are described in detail in the text.
(PDF)
