# Supplementary material for: SEED Servers: High-Performance Access to the SEED Genomes, Annotations, and Metabolic Models
Source: PLoS One. 2012 Oct 24;7(10):e48053. doi: 10.1371/journal.pone.0048053 (PMC3480482; doi:10.1371/journal.pone.0048053)
Supplement: Supporting Information S1 — Examples of programming using the SEED servers (coded in Perl). Eight application examples are provided, seven of which (1–4 and 6–8) are described in detail in the text. (PDF) [file pone.0048053.s001.pdf]

DOI: 10.1371/journal.pone.0048053

SEED Servers: High-Performance Access to the SEED Genomes, Annotations, and Metabolic Models

Supporting Information S1

Examples of programming using the SEED servers (coded in Perl)

Example Code 1:

URL: http://servers.theseed.org/sapling/server.cgi?code=server_paper_example1.pl

```
use strict;
use SAPserver;
use Data::Dumper;

# Example 1: ACH Data for a Protein
#
# This script takes a protein ID on the command line and
# finds all identifiers in the Sapling database that correspond
# to the identified protein (that is, they have the same amino
# acid sequence). For each identifier found, the following six
# columns will be output.
#
#   1. The identifier found.
#   2. The scientific name of the associated genome (if any).
#   3. 1 if we believe the identifier corresponds to the
#      exact gene identified by the input identifier, else
#      0. If the input identifier does not specify a
#      particular gene, this column will always be 0.
#   4. The functional assignment associated with the protein
#      ID.
#   5. The source of the assignment.
#   6. 1 if the assignment is considered expert, else 0.
#
# The data in columns (1), (4), (5), and (6) are provided
# automatically by the Sapling Server method "equiv_sequence".
# The item in column (3) is extracted via a call to the Sapling
# Server method "genome_names". The tricky part is the value in
# column (3). To make this determination, we make an initial call
# to the "equiv_precise" method to get identifiers for precisely-
# equivalent genes and put them into a hash. The value of
# column (3) is then determined by whether or not an
# identifier is in the precise-equivalence hash.

my $sapObject = SAPserver->new();
my $id = $ARGV[0];
if (! $id) {
    die "No protein ID specified.";
```

DOI: 10.1371/journal.pone.0048053

```
    } else {
        my %preciseHash;
        my $precise_assertions_list = $sapObject->equiv_precise_assertions(-ids
=> $id);
        $precise_assertions_list = $precise_assertions_list->{$id};
        if (@$precise_assertions_list > 0) {
          my $inputID = $id;
            for my $precise_assertion (@$precise_assertions_list) {
                my ($newID, $function, $source, $expert) = @$precise_assertion;
                $preciseHash{$newID} = 1;
            }
        }

        my $assertions = $sapObject->equiv_sequence_assertions(-ids => $id);
        $assertions = $assertions->{$id};
        if (@$assertions < 1) {
            print STDERR "No results found.\n";
        } else {
            for my $assertion (@$assertions) {
                my ($newID, $function, $source, $expert, $genomeName) =
@$assertion;
                $genomeName = '' if ! defined $genomeName;
                my $column3 = ($preciseHash{$newID} ? 1 : 0);
                print join("\t", $newID, $genomeName, $column3, $function,
$source,
                                 $expert) . "\n";
            }
        }
    }
```

DOI: 10.1371/journal.pone.0048053

Example Code 2:

URL: http://servers.theseed.org/sapling/server.cgi?code=server_paper_example2.pl

```
use strict;
use ANNOserver;

# Example 2: Metabolic Reconstruction for a Complete Prokaryotic Genome

# This script reads a set of gene ID / function pairs and produces a table of the
# subsystems that can be identified. The pairs are taken from the standard
# input. The input is in the form of a tab-delimited file, each record
# containing a gene ID followed by the function assigned to the gene. The output
# file is also tab-delimited. Each output record will contain a subsystem ID,
# its variant code, the relevant functional role, and its gene ID.
#
# By passing in all of the functional roles for a particular genome, you can
# use this program to find roles that may imply missing gene calls.


my $asObject = ANNOserver->new();
my @pairs;
while () {
    chomp;
    my ($id, $role) = split(/\t/, $_);
    push @pairs, [$role, $id];
}

my $reconstruction = $asObject->metabolic_reconstruction(-roles => \@pairs);
for my $record (@$reconstruction) {
    my ($variantID, $role, $id) = @$record;
    my ($subsysID, $code) = $variantID =~ /^(.+):(.+)$/;
    print join("\t", $subsysID, $code, $role, $id) . "\n";
}
```

DOI: 10.1371/journal.pone.0048053

Example Code 3:

URL: http://servers.theseed.org/sapling/server.cgi?code=server_paper_example3.pl

```
use strict;
use SAPserver;
use Data::Dumper;

my ($geneID,$max_distance);
my $usage = "usage: server_paper_example3 GeneID MaxDistance";
(
 ($geneID       = shift @ARGV) &&
 ($max_distance = shift @ARGV)
 )
    || die $usage;

my $sapObject = SAPserver->new();
my $geneLocH  = $sapObject->fid_locations(-ids => [$geneID]);
my $geneLoc   = $geneLocH->{$geneID}->[0];
if ($geneLoc =~ /^(\S+)_(\d+)([+-])(\d+)/)  # retrieve an encoded location
{
    my($contig,$beg,$strand,$length) = ($1,$2,$3,$4);
    my ($left,$right);
    if ($strand eq "+")
    {
      ($left,$right) = ($beg, $beg + ($length-1));
    }
    else
    {
      ($left,$right) = ($beg, $beg - ($length-1));
    }
    my $paddedLeft      = ($left > $max_distance) ? $left  - $max_distance :
1;
    my $paddedRight     = $right + $max_distance;
    my $sz              = ($paddedRight + 1) - $paddedLeft;
    my $region          = $contig . "_" . $paddedLeft . "+" . $sz;
    my $genesInRegionH  = $sapObject->genes_in_region(-locations =>
[$region],
                                                          -includeLocation =>
1);
    my $genesInRegion   = $genesInRegionH->{$region};
    foreach my $geneID2 (keys(%$genesInRegion)) {
        my $location = $genesInRegion->{$geneID2};
        $location =~ /^(\S+)_(\d+)([+-])(\d+)/;
      print "$geneID2\t$1\t$2\t$3\t$4\n";
    }
}
```

DOI: 10.1371/journal.pone.0048053

Example Code 4:

URL: http://servers.theseed.org/sapling/server.cgi?code=server_paper_example4.pl

```
#!/usr/bin/perl -w
use strict;
use SAPserver;
use COserver;
use SeedUtils;

my $sapObject = SAPserver->new();

my $genomeHash = $sapObject->all_genomes(-complete => 1);
for my $genome (keys %$genomeHash) {
    my $genomeName = $genomeHash->{$genome};
    my $geneHash = $sapObject->feature_assignments(-genome => $genome,
                                                   -type => 'peg');
    my @hypotheticalGenes = grep { &SeedUtils::hypo($geneHash->{$_}) } sort keys %$geneHash;
    my $couplingHash = $sapObject->conserved_in_neighborhood(-ids => \@hypotheticalGenes,
                                                             -hash => 1);
    for my $gene (@hypotheticalGenes) {
        my $couplingList = $couplingHash->{$gene};
        if (defined $couplingList) {
            my $subHash = $sapObject->ids_to_subsystems(-ids => [ map { $_->[1]} @$couplingList ]);
            my ($bestCoupler, $bestScore, $bestFunction) = (undef, 0, '');
            for my $coupling (@$couplingList) {
                my ($score, $coupler, $function) = @$coupling;
                if ($subHash->{$coupler} && $score > $bestScore) {
                    $bestCoupler = $coupler;
                    $bestScore = $score;
                    $bestFunction = $function;
                }
            }
            if (defined $bestCoupler) {
                print join("\t", $gene, $geneHash->{$gene}, $genome, $genomeName,
                                 $bestCoupler, $bestScore, $bestFunction) . "\n";
            }
        }
    }
}
```

DOI: 10.1371/journal.pone.0048053

Example Code 5:

URL: http://servers.theseed.org/sapling/server.cgi?code=server_paper_example5.pl

```perl
#!/usr/bin/perl -w
use strict;

use ANNOserver;

# Parms are FASTA input file name, genus, species.
# Produce geneID, type, contig, begin, strand, len on STDOUT.

my $annoObject = ANNOserver->new();
my ($file, $genus, $species) = @ARGV;

open FASTAIN, "<$file";
my $geneCalls = $annoObject->call_genes(\*FASTAIN);
# We throw away the FASTA string returned by call_genes.
my (undef, $geneList) = @$geneCalls;
for my $geneData (@$geneList) {
    my ($gene, $contig, $begin, $strand, $len) = @$geneData;
    print join("\t", $gene, 'peg', $contig, $begin, $strand, $len);
}
close FASTAIN;
open FASTAIN, "<$file";
my $rnaCalls = $annoObject->find_rnas(\*FASTAIN, $genus, $species, 'Bacteria');
# We throw away the FASTA string returned by find_rnas.
my (undef, $rnaList) = @$rnaCalls;
for my $rnaData (@$rnaList) {
    my ($rna, $contig, $begin, $strand, $len) = @$rnaData;
    print join("\t", $rna, 'rna', $contig, $begin, $strand, $len);
}
```

DOI: 10.1371/journal.pone.0048053

Example Code 6:

URL: http://servers.theseed.org/sapling/server.cgi?code=server_paper_example6.pl

```
#!/usr/bin/perl -w
use strict;

use ANNOserver;
use Data::Dumper;

# Reads FASTA protein sequences from STDIN.
# Produces sequence ID, role, genome set name, confidence
# If "-blast" is specified, uses BLAST option.

my $annoObject = ANNOserver->new();
my $blastOption = ($ARGV[0] && $ARGV[0] =~ /^-blast/i ? 1 : 0);

my $results = $annoObject->assign_function_to_prot(-input => \*STDIN,
                                                    -kmer => 8,
                                                    -scoreThreshold => 3,
                                                    -assignToAll => $blastOption);
while (my $data = $results->get_next()) {
    my ($id, $role, $genomeSet, undef, $hits) = @$data;
    # Only proceed if a role was found.
    if ($role) {
        $genomeSet = '' if ! defined $genomeSet;
        print join("\t", $id, $role, $genomeSet, $hits) . "\n";
    }
}
```

DOI: 10.1371/journal.pone.0048053

Example Code 7:

URL: http://servers.theseed.org/sapling/server.cgi?code=server_paper_example7.pl

```
#!/usr/bin/perl -w
use strict;
use FBAMODELserver;

# Reads the SEED genome-scale metabolic model of E. coli
# Runs flux variability analysis, flux balance analysis, and single gene knockout simulations in E. coli
# Prints all results to file

#1.) Creating perl object which interfaces with the SEED server API
my $fbaObject = FBAMODELserver->new();

#2.) Obtain a list of the reaction IDs in the E. coli model
my $reactionList = $fbaObject->get_reaction_id_list({id => "Seed83333.1"});

#3.) Obtain a list of the compound IDs in the E. coli model
my $compoundList = $fbaObject->get_compound_id_list({id => "Seed83333.1"});

#4.) Obtaining detailed data on E. coli reactions
my $reactionData = $fbaObject->get_reaction_data({"id" => $reactionList->{"Seed83333.1"},"model" => ["Seed83333.1"]});

#5.) Obtaining detailed data on E. coli compounds
my $compoundData = $fbaObject->get_compound_data({"id" => $compoundList->{"Seed83333.1"},"model" => ["Seed83333.1"]});

#6.) Running flux variability analysis on E. coli model while simulating growth in LB media
my $fvaOutput = $fbaObject->classify_model_entities({"parameters" => [{id=>"Seed83333.1",media=>"ArgonneLBMedia"}]});
#Placing fva output into a hash to simplify access
my $fvaHash;
for (my $i=0; $i < @{$fvaOutput->[0]->{entities}};$i++) {
      $fvaHash->{substr($fvaOutput->[0]->{entities}->[$i],0,8)} = $fvaOutput->[0]->{classes}->[$i];
}

#7.) Simulating growth of E. coli model in LB media
my $fbaOutput = $fbaObject->simulate_model_growth({"parameters" => [{id=>"Seed83333.1",media=>"Carbon-D-Glucose"}]});
#Placing fba output into a hash to simplify access
my $fbaHash;
for (my $i=0; $i < @{$fbaOutput->[0]->{entities}};$i++) {
      $fbaHash->{$fbaOutput->[0]->{entities}->[$i]} = $fbaOutput->[0]->{fluxes}->[$i];
}

#8.) Simulating single gene knockout in E. coli model during growth in LB media
my $KOOutput = $fbaObject->simulate_all_single_gene_knockout({"parameters" => [{id=>"Seed83333.1",media=>"ArgonneLBMedia"}]});

#9.) Printing all compound data to output file CompoundTbl.txt
```

DOI: 10.1371/journal.pone.0048053

```
my $columns = ["DATABASE","NAME","FORMULA","CHARGE","FVA class","FBA uptake flux"];
open (OUTPUT, ">CompoundTbl.txt");
print OUTPUT join("\t",@{$columns})."\n";
for (my $i=0; $i < @{$compoundList->{"Seed83333.1"}}; $i++) {
    for (my $j=0; $j < @{$columns}; $j++) {
        if ($j > 0) {
            print OUTPUT "\t";
        }
        if ($columns->[$j] eq "FVA class") {
            if (defined($fvaHash->{$compoundList->{"Seed83333.1"}->[$i]})) {
                print OUTPUT $fvaHash->{$compoundList->{"Seed83333.1"}->[$i]};
            }
        } elsif ($columns->[$j] eq "FBA uptake flux") {
            if (defined($fbaHash->{$compoundList->{"Seed83333.1"}->[$i]})) {
                print OUTPUT $fbaHash->{$compoundList->{"Seed83333.1"}->[$i]};
            }
        } else {
            if (defined($compoundData->{$compoundList->{"Seed83333.1"}->[$i]}->{$columns->[$j]})) {
                print OUTPUT join(", ",@{$compoundData->{$compoundList->{"Seed83333.1"}->[$i]}->{$columns->[$j]}});
            }
        }
    }
    print OUTPUT "\n";
}
close (OUTPUT);

#10.) Printing all reaction data to output file ReactionTbl.txt
$columns = ["DATABASE","NAME","EQUATION","Seed83333.1 COMPARTMENT","Seed83333.1 ASSOCIATED PEG","Seed83333.1 DIRECTIONALITY","FVA class","FBA flux"];
open (OUTPUT, ">ReactionTbl.txt");
print OUTPUT join("\t",@{$columns})."\n";
for (my $i=0; $i < @{$reactionList->{"Seed83333.1"}}; $i++) {
    for (my $j=0; $j < @{$columns}; $j++) {
        if ($j > 0) {
            print OUTPUT "\t";
        }
        if ($columns->[$j] =~ m/^Seed83333\.1\s(.+)/) {
            my $columnName = $1;
            if (defined($reactionData->{$reactionList->{"Seed83333.1"}->[$i]}->{"Seed83333.1"}->{$columnName})) {
                print OUTPUT join(" or ",@{$reactionData->{$reactionList->{"Seed83333.1"}->[$i]}->{"Seed83333.1"}->{$columnName}});
            }
        } elsif ($columns->[$j] eq "FVA class") {
            if (defined($fvaHash->{$reactionList->{"Seed83333.1"}->[$i]})) {
                print OUTPUT $fvaHash->{$reactionList->{"Seed83333.1"}->[$i]};
            }
        } elsif ($columns->[$j] eq "FBA flux") {
```

```
                    if (defined($fbaHash->{$reactionList->{"Seed83333.1"}->[$i]})) {
                          print OUTPUT $fbaHash->{$reactionList->{"Seed83333.1"}->[$i]};
                    }
              } else {
                    print OUTPUT $reactionData->{$reactionList->{"Seed83333.1"}->[$i]}->{$columns->[$j]}->[0];
              }
        }
        print OUTPUT "\n";
  }
  close (OUTPUT);
  #11.) Printing essential gene predictions to output file GeneTbl.txt
  open (OUTPUT, ">GeneTbl.txt");
  print OUTPUT "Predicted essential E. coli genes\n";
  for (my $i=0; $i < @{$KOOutput->[0]->{"essential genes"}}; $i++) {
        print OUTPUT $KOOutput->[0]->{"essential genes"}->[$i]."\n";
  }
  close (OUTPUT);
```

DOI: 10.1371/journal.pone.0048053

Example Code 8:

Short URL: http://bit.ly/server_paper_example8

This example includes two scripts:

(i) parse_uniprot.pl

URL: http://edwards-sdsu.cvs.sourceforge.net/viewvc/edwards-sdsu/bioinformatics/bin/parse_uniprot.pl?view=log

```
#__perl__

use strict;
use Digest::MD5;
use Data::Dumper;

# a simple uniprot parser because the parser in the version of bioperl  I have is broken.
# this is extremely simple, we are just printing out the id, accession number, go terms, md5 sum (so we can map to seed ids) and protein sequence

my $f = shift || die "Swiss Prot file?";
my $record;
my $inseq = 0;
# open the file, regardless of format
if ($f =~ /gz$/) {
        open(IN, "gunzip -c $f|") || die "Can't open a pipe to gunzip $f";
} elsif ($f =~ /zip$/) {
        open(IN, "unzip -p $f|") || die "can't open a pipe to unzip $f";
}       else {
        open(IN, $f) || die "can't open $f";
}

while (<IN>) {
        chomp;
        if (/^\/\/$/) {
                if ($record) {&output($record)};
                undef $record;
                $inseq=0;
                next;
        }
        if ($inseq) {
                s/^\s+//;
                $record->{'SQ'} .= $_;
                next;
        }
        m/^(\w\w)\s+(.*?)$/;
        my ($id, $data) = ($1, $2);
        if ($id eq "SQ") {$inseq = 1; next}
        push @{$record->{$id}}, $data;
}


sub output {
        my $rec = shift;
        #die Dumper($rec);
```

DOI: 10.1371/journal.pone.0048053

```
        my $trans = $rec->{'SQ'};
        $trans =~ s/\s//g;
        my $md5 = Digest::MD5::md5_hex(uc($trans));
        my $go = join("; ", grep {m/^GO/} @{$rec->{'DR'}});
        $rec->{'ID'}->[0] =~ m/^\s*(\S+)\s/;
        my $id = $1;

        foreach my $ac (map {split /\;\s*/, $_} @{$rec->{'AC'}}) {
                print join("\t", $id, $ac, $go, $md5, $trans), "\n";
        }
}
```

(ii) map_uniprot_to_seed.pl

URL: http://edwards-sdsu.cvs.sourceforge.net/viewvc/edwards-sdsu/bioinformatics/bin/map_uniprot_to_seed.pl?view=log

```
#__perl

use strict;
use SAPserver;
use Data::Dumper;
my $sap=new SAPserver;
use Getopt::Std;
my %opts;
getopts('f:n:', \%opts);

die <<EOF unless ($opts{f});
$0
-f filename to get
-n number of proteins to parse at once. The default is 1,000 proteins


This is a program to retrieve essential seed data (role, subsystems, and subsystem classifications) about a set of proteins. To map into the seed we map based on the MD5 sum of the uppercase protein sequence (just the protein sequence alone, and without any identifiers or stop symbol). We can then move from those ids to subsystems and roles.

This code uses the seed servers, and you can find more information about them at http://servers.theseed.org/ You will need to install the seed servers before starting, but that should install all the commands you need.

Basically, we make remote calls that retrieve data, and the calls are designed so that we can retrieve a bolus of data and compute on it. In this case, you can set how many pieces of data to send/receive with the -n option. I suggest a relatively large number, like 1000, since the delay is typically in retrieving the data and not in sending the data over the internet.

This will print the results out, and so you may need to redirect the output.

EOF


unless (defined $opts{n}) {$opts{n} = 1000}

```

DOI: 10.1371/journal.pone.0048053

```
# get the subsytem hierarchy. We only need this once, so getting it here will let us use it later
my $ssHash = $sap->all_subsystems(-usable => 1);

# counters and raw data
my $ids; my $count=0;
# open the file and iterate through every line
# this allows us to open gzipped or zipped files
if ($opts{f} =~ /gz$/) {
        open(IN, "gunzip -c $opts{f}|") || die "Can't open a pipe to gunzip $opts{f}";
} elsif ($opts{f} =~ /zip$/) {
        open(IN, "unzip -p $opts{f}|") || die "can't open a pipe to unzip $opts{f}";
}       else {
        open(IN, $opts{f}) || die "can't open $opts{f}";
}

while (<IN>) {
        chomp;
        # this is the input data
        my ($id, $ac, $go, $md5, $trans)= split /\t/;
        # store it and make a call with lots of data
        $ids->{$md5}=[$id, $ac, $go, $md5, $trans];
        $count++;
        if ($count == $opts{n}) {
                # we need to make the call and print the information out
                # don't forget to reset our counters.
                &get_data($ids);
                $count=0;
                $ids={};
        }
}
close IN;


sub get_data {
        my $ids = shift;

        # convert the md5 sums to fig ids
        # all of the subsequent calls require fig ids
        my $idHash = $sap->proteins_to_fids({-prots=>[keys %$ids]});
        my $figids;
        foreach my $id (keys %$idHash) {
                map {$figids->{$_}=1} @{$idHash->{$id}};
        }

        # get the subsystems for each proteins
        my $subsysHash = $sap->ids_to_subsystems({-ids=>[keys %$figids], -usable=>1});

        # now print out everything
        # we need to iterate through the md5s and the fid ids to merge everything together
        foreach my $md5 (keys %$ids) {
                foreach my $fid (@{$idHash->{$md5}}) {
                        # get the functional role and subsystem for this protein
```

DOI: 10.1371/journal.pone.0048053

```
					# recall that this is a one:many relationship
					foreach my $tple (@{$subsysHash->{$fid}}) {
						my ($role ,$ss)=@$tple;
						# in case the classification isn't defined, we'll make it null
						if (!defined $ssHash->{$ss}) {$ssHash->{$ss}=['', ['', '']]}
						# print everything out
						print join("\t", @{$ids->{$md5}}, $fid, $role, $ss, @{$ssHash->{$ss}->[1]}), "\n";
					}
				}
		}
	}
```
